# On Hemorrhage

**Published:** 1853-06

**Authors:** M. Pagliari


From the Gazette Medicale.

*On Hemorrhage. On a New Styptic.* By M. PAGLIARI.

THIS styptic forms a subject of a long communication to the Academie des Sciences, by C. Sedillot. Its composition is as follows: eight ounces of balsam of benzoin, one pound of sulphate of alumina and potass, and ten pounds of common water are boiled for six hours in a glazed earthen vessel, care being taken to add fresh quantities of *boiling* water, so as to supply the loss in evaporation, and to stir continually. At the end of this time the supernatant liquid is separated from the undissolved benzoin, which has lost its odor and inflammability, and filtered and preserved in glass bottles. The liquid thus obtained, is limpid, and of the color of champagne; its taste lightly stypic, and its odor pleasant and aromatic, and when evaporated it leaves a transparent deposit on the sides of the vessel.

The styptic properties of this preparation seem very remarkable. A single drop immediately coagulates a cupping-glassful of blood, and a larger quantity (equal proportions) converts the blood into a firm and resisting solid. Applied to a wound, the hemorrhage ceases almost immediately, in consequence, as it would seem, of the formation of such clots upon all the orifices of the bleeding vessels. The application also produces no irritation and inconvenience, nor does it interfere in any way with the process of cicatrization.

The cases given are well authenticated, and the results such as to leave no doubt as to the valuable styptic properties of the preparation. There are cases of obstinate primary and secondary hemorrhages, after surgical operations; one from a severe cut in the finger; some from the extraction of a tooth. In these cases a piece of lint was soaked in the styptic and bound upon the wound.

